# Supplementary material for: Broccoli aptamer allows quantitative transcription regulation studies in vitro
Source: PLoS One. 2024 Jun 13;19(6):e0304677. doi: 10.1371/journal.pone.0304677 (PMC11175446; doi:10.1371/journal.pone.0304677)
Supplement: S1 Text — (PDF) [file pone.0304677.s001.pdf]

S1. Derivation of a master curve for *lac* repression.

When calculating the fold change in a scenario with repressors, we have two unknown variables λ_r and λ_p (Eq (1)). In the weak promoter limit ($\lambda_p << 1$) Eq (1) can be simplified and will contain only λ_r as the unknown parameter. This makes it possible to collapse various data sets onto one master curve, which does not depend on variations of the polymerase. In our case, we wanted to include information about the polymerase in a master curve in a similar fashion.

$$\text{fold change} = \frac{\theta_s(\lambda_p, \lambda_r)}{\theta_s(\lambda_p, 0)} = \frac{1 + \lambda_p e^{-\beta\epsilon_{p,s}}}{1 + \lambda_p e^{-\beta\epsilon_{p,s}} + \lambda_r e^{-\beta\epsilon_{r,s}}} \tag{1}$$

By defining $\lambda*$ as a function of both λ_p and λ_r, we compose a new expression for the fold change (Eq (2) right side). We then equate this to Eq (1), which yields $\lambda^* = \frac{\lambda_r}{1+\lambda_p e^{-\beta\epsilon_{p,s}}}$.

$$\frac{1 + \lambda_p e^{-\beta\epsilon_{p,s}}}{1 + \lambda_p e^{-\beta\epsilon_{p,s}} + \lambda_r e^{-\beta\epsilon_{r,s}}} = \frac{1}{1 + \lambda^*(\lambda_p, \lambda_r)} \tag{2}$$

With this definition, we can plot $\lambda^* e^{-\beta\epsilon_{p,s}}$ against the new definition for the fold change, as shown in Fig 3 of the main text.
