# Supplementary material for: Broccoli aptamer allows quantitative transcription regulation studies in vitro
Source: PLoS One. 2024 Jun 13;19(6):e0304677. doi: 10.1371/journal.pone.0304677 (PMC11175446; doi:10.1371/journal.pone.0304677)
Supplement: S2 Text — (PDF) [file pone.0304677.s002.pdf]

S2. Folding delays of the Broccoli-aptamer

We can quantify the transcription rate by measuring the fluorescent signal of our samples over time because the transcript is the Broccoli aptamer, a 105-nucleotide RNA sequence (B_i) that binds to the small molecular dye DFHBI-1T (D) to form a fluorescent RNA-dye complex (DB_a). For a constant RNA production rate

$$RNAP + T \xrightarrow{k_p} RNAP + T + B_i, \tag{1}$$

one would expect the fluorescent signal to increase linearly with time until the transcript concentration approaches the dye concentration and the signal saturates. We do indeed observe a linear regime followed by saturation, but the maximum rate of fluorescence increase is only reached after a lag time of approximately half an hour in all experiments.

We hypothesize that the reason for this initial delay is not that the RNA production itself is delayed, but that the fluorescent signal lags behind RNA production because the transcript needs to fold and bind to DFHBI-1T before an increase in fluorescent signal is observed. We do not *a priori* know which of the two processes—the folding of inactive Broccoli into its active confirmation ($B_i \rightarrow B_a$) , or the binding of the active Broccoli conformer with DFHBI-1T to form the fluorescent RNA-dye complex ($B_a + D \rightarrow DB_a$)—is rate limiting. The two processes could also occur cooperatively. Therefore we model the conversion of inactive B_i to the fluorescent complex as a single reaction with a rate constant k_r that is independent of molecular details of the reaction steps:

$$B_i + D \xrightarrow{k_f} DB_a \tag{2}$$

In our model B_i represents the set of all Broccoli conformations except for the conformation that binds to the dye and all Broccoli that is not in an "inactive conformation" is bound to the dye.

To find the time-dependent concentration of $DB_a(t)$, which provides the fluorescent signal, we first consider the total concentration of Broccoli $c_{Btot}(t) = c_{Bi}(t) + c_{DBa}(t)$. $c_{Btot}(t)$ increases linearly with time as $c_{Btot}(t) = k_p t$, where k_p (in units M/s) sets the rate and depends on the concentrations of the template, RNA polymerase, non-specific DNA, and repressor molecules. The concentration of B_i is increased by transcription and decreased by the reaction with the dye: (1) and (2):

$$\frac{dc_{Bi}}{dt} = k_p - k_f c_{Bi} \tag{3}$$

We assumed that the binding of Broccoli to the dye is a first order reaction with rate constant k_f. This is a reasonable assumption when the dye concentration far exceeds the RNA concentration.

Solving differential Eq (3), we find $c_{Bi}(t) = \frac{k_p}{k_f}\left(1 - e^{-k_f t}\right)$, which—combined with mass conservation—gives

$$c_{DBa}(t) = k_p \left(t - \frac{1 - e^{-k_f t}}{kf} \right) \tag{4}$$

In the long time limit ($t \gg 1/k_f$) $c_{DBa} \approx k_p t$, so that the fluorescent signal is a good measure of the transcription rate. At short times ($t \ll 1/k_f$) a Taylor expansion in $k_f t$ up to the second term gives $c_{DBa} \approx \frac{1}{2} k_p k_f t^2$, indicating that the fluorescence increases quadratically with time, consistent with our observations.

Taken together our model indicates that the shape of our fluorescent curves is consistent with the scenario where fluorescence lags behind transcription due to slow folding of the transcript. Under all conditions the transition from the quadratic to the linear regime occurs after a delay on the order of 10^3 seconds, which implies a folding rate $k_f = 10^{-3} s^{-1}$. This time is consistent with observations by Filonov *et al.* who observed a recovery of fluorescence in 10 to 15 minutes after denaturing Broccoli with Urea and allowing it to refold in presence of DFHBI-1T.[1]

References

[1] Filonov GS, Kam CW, Song W, Jaffrey SR. In-Gel Imaging of RNA Processing Using Broccoli Reveals Optimal Aptamer Expression Strategies; p. 29.
