# Supplementary material for: Broccoli aptamer allows quantitative transcription regulation studies in vitro
Source: PLoS One. 2024 Jun 13;19(6):e0304677. doi: 10.1371/journal.pone.0304677 (PMC11175446; doi:10.1371/journal.pone.0304677)
Supplement: S3 Text — (PDF) [file pone.0304677.s003.pdf]

S3. Multi-chemical equilibrium model for tetrameric repression

To rule out the possibility of DNA-bridges formed by tetrameric repressors, which would lead to different assumptions in the model, we set up a multi-chemical model that accounts for bridging of DNA fragments by tetrameric repressors (Fig S1A). We assume three possible equilibria for DNA.

$$\begin{aligned} D + P &\rightleftharpoons DP \\ D + R &\rightleftharpoons DR \\ 2D + R &\rightleftharpoons D_2R \end{aligned} \tag{1}$$

where DP is the polymerase bound to a promoter, DR is a repressor bound to a DNA fragment, and D_2R is a repressor bound to two DNA fragments. We define from Eq (1) the following equilibrium constants

$$\begin{aligned} k_P &= \frac{[DP]}{[D][P]} = e^{-\beta\epsilon_P} \\ k_1 &= \frac{[DR]}{[D][R]} = e^{-\beta\epsilon_R} \\ k_2 &= \frac{[D_2R]}{[D]^2[R]} \approx k_1^2 = e^{-2\beta\epsilon_R} \end{aligned} \tag{2}$$

From the definitions in Eq (2), we establish mass balances for the polymerase, repressor and DNA assuming a fixed total concentration of each of these components.

$$\begin{aligned} [D_t] &= [D] + [DP] + [DR] + 2[D_2R] \\ [P_t] &= [P] + [DP] \\ [R_t] &= [R] + [DR] + [D_2R] \end{aligned} \tag{3}$$

These can then be rewritten using the equilibrium constants as defined earlier.

$$\begin{aligned} [D_t] &= [D] + k_P[D][P] + k_1[D][R] + 2k_1^2[D][R] \\ [P_t] &= [P] + k_P[D][P] + k_P[D][P] \\ [R_t] &= [R] + k_1[D][R] + k_1[D][R] + k_1^2[D]^2[R] \end{aligned} \tag{4}$$

We define the fold change as fold change $= \frac{\theta_P}{\theta_P(R=0)} = \frac{([D_P]/[D_t])}{([D_P]/[D_t])_{R=0}}$ and the occupation by the repressor as $\theta_R = \frac{[DR]+[D_2R]}{[D_t]} = \frac{k_1[D][R](1+K_1[D])}{[D_t]}$. In Fig S1B, the curves for θ_R for various DNA concentrations show that a plateau appears at sufficiently high DNA concentrations (purple line). Even though the repressor concentration increases, the fraction of occupied DNA sites remains constant. This happens when one tetramer bound to two DNA fragments gets replaced by two tetramers bound to single DNA fragments. The higher the DNA concentration, the stronger this plateauing effect.

However, the DNA concentration we are working at (10 nM) does not show this behaviour and we thus conclude that to a good approximation, all the DNA is bound to individual tetramers without bridging.

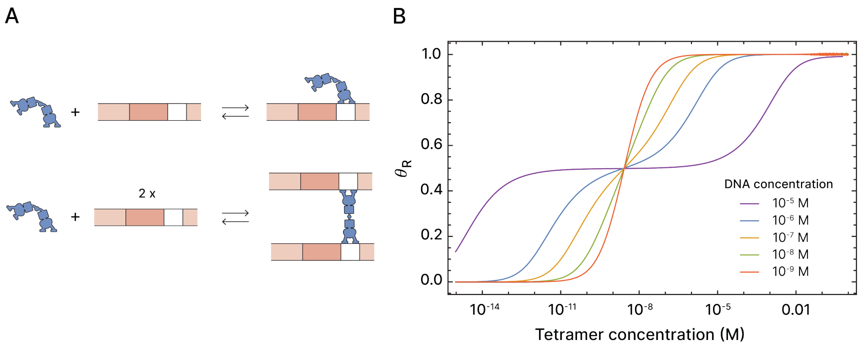

Fig. S1 Bridging by *lac* tetramers. A: Schematic drawing of the different ways DNA molecules can be occupied by tetrameric repressors. B: The fraction of DNA bound to a single repressor as a function of tetramer concentration, shown for various DNA concentrations. At $\theta_R = 0.5$, half of the DNA is bound in bridged fashion, and half is bound to a single tetramer.
