# Supplementary material for: Broccoli aptamer allows quantitative transcription regulation studies in vitro
Source: PLoS One. 2024 Jun 13;19(6):e0304677. doi: 10.1371/journal.pone.0304677 (PMC11175446; doi:10.1371/journal.pone.0304677)
Supplement: S4 Text — (PDF) [file pone.0304677.s004.pdf]

S4.Template controls

To show that the transcription of our DNA template is controlled by the T7 promoter, we performed transcription assays using DNA templates with and without a T7 promoter. The results are shown in Fig S2A, where the difference in transcription rate can be observed between DNA templates with and without a promoter. In absence of a promoter, we only see a very low transcription rate compared to a regular template. Additionally, we show that the *lac* repressor was able to effectively repress transcription only when the O1 operator was located directly downstream of the T7 promoter. For DNA templates that did not contain a O1 operator, where the T7 promoter was directly followed by the Broccoli sequence, the presence of the *lac* repressor did not lead to repression. This can be seen in Fig S2B, which shows the fold change as a results of the presence of the repressor. When the binding site is absent, addition of repressors will not lead to repression. Concentrations of the all reaction components in these experiments were similar to those in other typical transcription reactions.

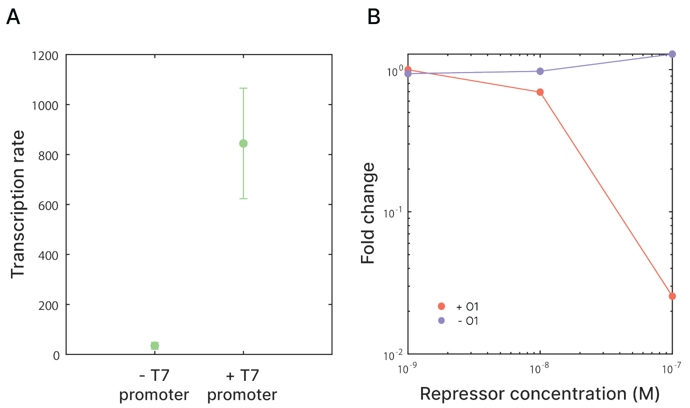

Fig. S2 Control experiments. A: Transcription rates for DNA fragments with and without a T7 promoter upstream of the Broccoli sequence. B: Repression by the *lac* repressor with and without an O1 binding site between the T7 promoter and the Broccoli sequence.
