# Supplementary material for: Broccoli aptamer allows quantitative transcription regulation studies in vitro
Source: PLoS One. 2024 Jun 13;19(6):e0304677. doi: 10.1371/journal.pone.0304677 (PMC11175446; doi:10.1371/journal.pone.0304677)
Supplement: S1 Table — (PDF) [file pone.0304677.s005.pdf]

S5. DNA sequences

Transcription sequences (5′ → 3′)	
2xF30-Broccoli	TTGCCATGTGTATGTGGGAGACGGTCGGGTCCAGATATTCGTATCTGTCGAGTAGAGTGTGGGCTCCCACATACTCTGATGATCCTTCGGGATCATTCATGGCAA
T7 promoter	TAATACGACTAACTATAGG
O1 operator	AATTGTGAGCGGATAACAATT
Transcription template	TCGTTGCGTTACACACACAAAAAACCAACACACATCCATCTTCGATGGATAGCGATTTTATTATCTAACTGCTGATCGAGTGTAGCCAGATCTTAATACGACTAACTATAGGCAATTGTGAGCGGATAACAATTCTAGATTGCCATGTGTATGTGGGAGACGGTCGGGTCCAGATATTCGTATCTGTCGAGTAGAGTGTGGGCTCCCACATACTCTGATGATCCTTCGGGATCATTCATGGCAAGGATCCG
**Primers and inserts (5′ → 3′)**	
OGP-F1 (forward)	TCGTTGCGTTACACACAC
Broc-rev (reverse)	CGGATCCTTGCCATGAAT
T7 promoter + O1 operator forward	GATCTTAATACGACTCACTATAGGCAATTGTGAGCGGATAACAATT
T7 promoter + O1 operator reverse	CTAGAATTGTTATCCGCTCACAATTGCCTATAGTGAGTCGTATTAA
