# Supplementary material for: Broccoli aptamer allows quantitative transcription regulation studies in vitro
Source: PLoS One. 2024 Jun 13;19(6):e0304677. doi: 10.1371/journal.pone.0304677 (PMC11175446; doi:10.1371/journal.pone.0304677)
Supplement: Attachment — Submitted filename: response_to_reviewers.docx [file pone.0304677.s007.docx]

Dear Dr. Liu,

Thank you for forwarding the reviewer comments on our manuscript entitled “Broccoli aptamer allows quantitative transcription regulation studies *in vitro”,* PONE-D-24-06193.

We were pleased to read the overall positive remarks of the reviewers and appreciate their constructive comments, suggestions and questions. Upon going through the comments, we realized that during the submission process, apparently the first paragraph of our introduction in the submitted manuscript has accidentally disappeared. This might explain some of the remarks made by the reviewers. We naturally included it in the revised manuscript, which will hopefully give the reader a better feel for the context we place our research in. Furthermore, we added some extra sentences to explain the term fugacity properly, as was rightfully suggested by one of the reviewers. Some experimental procedures were also clarified in the manuscript following comments from the reviewers, and we included more references to existing literature. We extended our speculation on potential applications of this method for future studies in response to the reviewers and expanded that part in the Discussion. Finally, as requested, the updated financial disclosure statement regarding the role of the funders: “The funders had no role in study design, data collection and analysis, decision to publish, or preparation of the manuscript." Regarding the supplementary information, we included everything in separate documents that include the figures. With these and other revisions as described in detail below, we hope our manuscript is acceptable for publication in PLOS One.

Yours sincerely,

On behalf of all authors,

Amanda van der Sijs

**Comments to the Author**

1. Is the manuscript technically sound, and do the data support the conclusions?

The manuscript must describe a technically sound piece of scientific research with data that supports the conclusions. Experiments must have been conducted rigorously, with appropriate controls, replication, and sample sizes. The conclusions must be drawn appropriately based on the data presented.

Reviewer #1: Partly

Reviewer #2: Yes

Reviewer #3: Yes

Reviewer #4: Yes

2. Has the statistical analysis been performed appropriately and rigorously?

Reviewer #1: Yes

Reviewer #2: Yes

Reviewer #3: Yes

Reviewer #4: Yes

3. Have the authors made all data underlying the findings in their manuscript fully available?

The [PLOS Data policy](http://www.plosone.org/static/policies.action#sharing) requires authors to make all data underlying the findings described in their manuscript fully available without restriction, with rare exception (please refer to the Data Availability Statement in the manuscript PDF file). The data should be provided as part of the manuscript or its supporting information, or deposited to a public repository. For example, in addition to summary statistics, the data points behind means, medians and variance measures should be available. If there are restrictions on publicly sharing data—e.g. participant privacy or use of data from a third party—those must be specified.

Reviewer #1: Yes

Reviewer #2: Yes

Reviewer #3: Yes

Reviewer #4: No

4. Is the manuscript presented in an intelligible fashion and written in standard English?

PLOS ONE does not copyedit accepted manuscripts, so the language in submitted articles must be clear, correct, and unambiguous. Any typographical or grammatical errors should be corrected at revision, so please note any specific errors here.

Reviewer #1: Yes

Reviewer #2: Yes

Reviewer #3: Yes

Reviewer #4: Yes

5. Review Comments to the Author

**Reviewer #1**: The manuscript presents detailed description of in vitro transcription depended on the concentration of a repressor and an enzyme. This description is logic and well-written, however, the coincience of the experiment and the model is not sufficient. The model does not predict the RNAP-dependent differences in curves that are clearly seen in Figures 3 and 4. This obstacle indicates some disfunction in the model. I suggest its revision an adding some other step.

Reply: It is not clear to us to exactly what difference the reviewer refers to. We agree that in Fig. 3 there is a slight difference between experiments and theory for the dimers. As we explain in the text, there could be several reasons for that . In the rest of Fig, 3 and in Fig. 4, the agreement between experiments and theory is excellent.

Changes in manuscript: Added to section Repression using LacI “Variability between protein batches might account for the differences we observed.”

Also it is not obvious what is the scientific novelty of the manuscript. Broccoli aptamer is a known tool for transcription visualization in cells. The description of transcription process was also performed a lot of times. It is unclear what new information can we extract from this research.

Reply: As we mention in the Introduction, Broccoli aptamers have indeed been used frequently in in vivo transcription visualization. However, the focus of our research is on its potential for quantitative studies in vitro. In vivo setups do not always allow for independent variation of the variables that control transcription, an important example being the concentration of non-specific DNA that we address in this manuscript in Results, Nonspecific DNA can effectively act as a repressor.

**Reviewer #2:** The authors present a quantitative in vitro transcription assay using RNA aptamers coupled to derivations of thermodynamic models of transcription. The approach is quite straightforward and the calculations are rigorous, which is welcome in the field. The results are not revolutionary compared to other available methods, but the authors show that the method follows expected quantitative predictions, with the advantages of fluorescence. They show an interesting example of repression by competitive binding of RNAP.

I have a few remarks regarding clarity and formatting.

1. The introduction contains a whole derivation that should not belong to the introduction (l 47 to 83). It interrupts the presentation of the paper. Consider moving that derivation to the Methods section, or to Results if you consider is as a new theoretical framework that accompanies the data.

Reply: We thank the reviewer for sharing their view on this section of the manuscript. We consider the theoretical framework essential for the reader to understand and interpret the results, hence our choice to place this section in the introduction. We dedicate a separate paragraph (3. Thermodynamic model for transcription regulation) so that it can easily be skipped by those who are familiar with the theoretical background. We feel that moving this section to the Methods section will lead to the theoretical framework being overlooked, making the Results section hard to interpret.

2. The calculation is derived in the grand canonical ensemble using "fugacities". The theory seems fine to me. But this framework is hard to follow for anyone who is not an expert in chemical thermodynamics, especially in the gene regulation community. It is the authors' choice to choose their readership. Please at least give the definition of fugacity. Other theoretical models have expressed the expression as a function of RNAP/regulator concentration, using a different ensemble. As a theoretician, I appreciate the rigor exhibited by the authors of this paper; although as a computational biologist focusing on regulation, I do appreciate being able to understand the main equations in terms of experimentally accessible variables such as concentrations, if this does not prevent a rigorous analysis.

Reply: We apologize that we did not properly explain the definition of fugacity and thank the reviewer for pointing this out. We added a more extensive explanation. Regarding the ability to understand the equations in terms of experimentally accessible variables, we would like to point out equations 4 and 5, which include the concentrations of both the DNA and proteins. These mass balances allow us to find the fugacities of the proteins (less accessible variables), and we feel that this coupling is sufficient to help the reader to relate the other equations to experimentally accessible variables.

Changes in manuscript: Added to section Thermodynamic model for transcription regulation: “Formally the fugacity is a Lagrange multiplier that couples to the constraint that total concentration (of RNAP, TF) is conserved. The physical interpretation of fugacity is the free, that is, unbounded, concentration of RNAP, TF in solution. In our framework, the fugacities are obtained by implication of mass balance in Eqs. (4),(5).”

3. l 178: "which depends on both the rate of RNA synthesis and the rate of aptamer folding". This sentence does not mention the possible role of fluorophore binding, although it is included in the calculation S2.

Reply: We thank the reviewer for noticing this and we now added an explanation to that sentence to clarify it.

Changes in manuscript: Added to section Folding delays: “which depends on both the rate of RNA synthesis and the rate of formation of the fluorescent RNA-dye complex. The latter consists of the folding of the RNA and the binding to the dye. As we do not know which of these processes is rate limiting, we model the conversion of inactive Broccoli RNA to the fluorescent complex as a single reaction with rate k_f_.”

4. Delay because of folding. I was surprised by the very slow folding, although the authors mention a comparable reported value. This is a drawback, as mentioned in the Discussion. Maybe the authors should discuss if the in vivo timescales are comparable, and/or if the method seems also applicable in vivo (in terms of fluorescence intensity).

Reply: This is indeed an important restriction of the method. In recent work by Purhonen et al. (2024), a similar delay time can be observed. Assuming that the reviewer is referring to the transcription timescales, we see no reason to expect that the timescale in vivo is different from our findings . If anything, we expect the timescales in vivo to be longer than those in vitro, which would make the timescale of the folding delay less of a problem. Applications of Broccoli for in vivo visualization purposes has been reported by Li et al. (2020), Zinksie et al. (2018), and Catala et al. (2023) describe F30-2xdBroccoli as a reporter for gene expression in E. coli. Note that in these works, no time scales are being mentioned. We added these references to the introduction.

Changes in manuscript:
Added to section Introduction, Broccoli aptamer: “*In vivo*, Broccoli has been applied to visualize RNA in cells and as a gene expression reporter. (Zinskie et al. 2018, Catala et al. 2023, Li et al. 2020).”

Added to section Folding delays: “In more recent work by Purhonen *et al.* (2024)*,* a similar delay in the fluorescent signal can be observed in their transcription experiments.”

5. l 216: "The key is that...". I don't see what key; the concentrations of RNAP and regulator may be (and in fact are in the cell) hugely different (maybe 1000-fold); so a difference in binding free energy is not conclusive in itself, is it?

Reply: We thank the reviewer for noticing this. This sentence is indeed out of place and should have been removed before submitting the manuscript. This difference in itself is not conclusive and we are not aiming at concentration differences.

Changes in manuscript: removed from section Repression using LacI “The key is that the difference between the RNAP and the repressor binding energy is approximately 5 kBT.”

6. Fig. 4: The authors mention the concentration values of competitive DNA, but (maybe I missed in the text) the concentration of promoter DNA used. I would assume the competition depends on the ratio promoter/competitor, so this seems important to me but is not mentioned in the Results paragraph nor the figure legend. Or let me know if I am mistaken. Also, I assume that the binding free energy of competitor DNA depends strongly on its sequence, presence of promoter-like sequences, etc? The authors only consider one competitive sequence, so they might warn the reader that the inferred value is specific to this experiment.

Reply: The concentration of promoter DNA is indeed important and was accidentally omitted, for which we apologize. We added the concentrations to the methods section. Regarding the competitive sequences of nonspecific DNA, the binding free energy does indeed depend on the sequence. We used salmon sperm DNA, which contains negligible amounts of T7 promoter-like sequences. In the 2.8 Gb genome, a BLAST search found 119 matches with the 19 nucleotide long T7 promoter, of which only 3 full length alignments. We now explicitly mention that the results we present are specific to this type of DNA. However, we expect the trend for all non-specific DNA is very similar to the results in Fig.4 and believe this is representative for nonspecific DNA in cells.

Changes in manuscript: Added to section Methods, In vitro transcription: “The concentration of DNA template containing the promoter, operator and Broccoli-sequence was 10 nM in all experiments.” Added to a sentence in section Results, In vitro transcription rates are proportional to promoter occupancy: “For these experiments, we used DNA template consisting of the T7 promoter directly followed by the Broccoli sequence *at a concentration of 10 nM*.”

Added to section Nonspecific DNA can effectively act as a repressor: “These experiments were performed using salmon sperm DNA, which contains a negligible number of promoter-like sequences specific to T7 RNAP.” And: “While these numbers are specific to the DNA used in our experiments, we expect that all types of non-specific DNA will follow the global trend in Fig.4.”

**Reviewer #3**: The paper presents an investigation into transcription regulation in vitro using the fluorescent Broccoli aptamer, focusing on the relationship between RNAP promoter occupancy and transcription rates, the effect of folding delays on transcription rate measurement, the repression mechanism via the LacI repressor, and the impact of nonspecific DNA binding on transcription activity.

While the experimental setup and the data presented are methodologically sound, providing quantitative insights into transcription regulation and the role of nonspecific DNA binding, the paper seems to reiterate findings that align closely with established models and previous studies. The confirmation of proportional relationships between transcription rates and promoter occupancy by RNAP, the quantification of repression mechanisms using LacI, and the influence of nonspecific DNA on transcriptional activity do not deviate significantly from expected outcomes based on existing literature. The data on the influence of nonspecific DNA on transcriptional activity is especially weak.

Reply: Our study indeed reaffirms findings from previous research, and we think it is important to validate existing results in order to establish the utility and robustness of the method with Broccoli as fluorescent aptamer. Although the experiments by themselves do indeed not directly lead to breakthrough findings, we feel they provide a solid basis for future studies. We do not understand the point that “the influence of nonspecific DNA is especially weak", as the reviewer provides no explanation for this statement. In Fig. 4 we provide quantitative evidence that the effect is significant and should be taken into account in transcription regulation studies.

One of the paper's main contributions is its quantitative approach towards understanding the dynamics of transcription regulation in a controlled in vitro setting, using a novel fluorescence-based method. However, the novel insights for researchers, especially those looking for breakthrough findings or transformative concepts in the field of transcription regulation, may seem limited. The validation of existing models and the reaffirmation of known regulatory mechanisms, while valuable, do not present a significant leap forward in our understanding of transcriptional regulation complexities.

Reply: we addressed this comment in the previous reply.

Furthermore, the paper discusses the limitations associated with the use of the Broccoli aptamer, particularly the folding delay impacting the immediacy of transcription rate measurements, which suggests that while the method is innovative, it might not be universally applicable for all types of transcriptional studies, especially those requiring real-time analysis.

Reply: we agree that the method is not universally applicable, mainly because of the relatively slow folding time. This makes the method not the best choice in studies with fast concentration variations and steep spatial gradients. However, in all other situations, as we show in this manuscript, the Broccoli method can be confidentially applied to address quantitative questions regarding transcription regulation.

In conclusion, the paper provides a solid experimental framework and a detailed quantitative analysis of transcription regulation mechanisms in vitro. It reaffirms established models and offers precise measurements that could serve as a reference for future studies. However, for a research community keen on groundbreaking discoveries and novel insights into the regulation of gene expression, the findings may appear as incremental advancements rather than substantial leaps forward. Future work that builds on these methodologies to explore uncharted territories in gene regulation or introduces innovative experimental designs to capture the dynamic nature of transcriptional regulation might be more impactful for advancing the field.

Reply: we agree we “only” provide incremental advancement. See next points for more detail and possible long(er) term implications of our work.

Main questions:
1. Could the authors elaborate on the novel contributions of their study, especially in the context of how their findings significantly advance our understanding of transcription regulation beyond validating existing models? How does the use of the fluorescent Broccoli aptamer specifically contribute to new insights that were previously unattainable?

Reply: To our knowledge, the direct influence of nonspecific DNA on transcription has not been systematically studied before. Such experiments are not possible *in vivo*, as one cannot systematically vary the number of accessible base pairs in a cell. The use of the Broccoli aptamer has thus been crucial in exploring this effect in its most basic form, and in future studies in more complex environments.

Changes in manuscript: see Changes in manuscript as a response to the next question.

2. The authors have provided a quantitative framework for understanding transcription regulation. Can they discuss how their quantitative measurements compare with the predictions of existing models?

Reply: We can distinguish roughly two approaches to describe transcription regulation: thermodynamic and kinetic models. The thermodynamic model we present in the manuscript is in fact an existing model, that has been verified to work for certain in vivo scenarios.(Landman et al. 2019, Weinert et al. 2014) We use this framework to interpret the findings of our experiments, and explore how far we can take these models to describe different experimental results. In future experiments, we might find that these models are not suitable to describe all regulatory phenomena. If so, we will take a different approach towards modelling these results. However, in the experiments performed for this paper, we see no reason to apply a transcription model based on kinetics. Due to the folding time of Broccoli, fast dynamics are outside the scope of what we are able to measure. Hence our choice to use thermodynamic models, that adequately describe steady-state mean levels of transcription.

Changes in manuscript for questions 1 and 2: “Previous studies using the thermodynamic transcription regulation model have successfully taken the influence of nonspecific DNA on regulatory outcomes into account. However, the effect of changes in the availability of nonspecific DNA for transcription factors to bind to have not been included in these studies. Due to cellular processes such as replication and nucleosomal (re)organisation, the amount of DNA accessible to transcription factors can vary. It is predicted that this effect is nontrivial; even slight changes in transcription factor concentration and variations in the DNA available to bind to might lead to substantial differences in transcriptional outcomes. Our method enables systematically studying this.”

3. The manuscript mentions the folding delays associated with the Broccoli aptamer and its impact on transcription rate measurements. How do the authors propose to mitigate these limitations in future studies? Are there alternative approaches or improvements to the Broccoli aptamer method that could minimize these delays?

Reply: As we see it, the delay is inherent in the method and limits the applicability in vivo to situations where transcription is relatively slow and concentration gradients (of mRNA) are weak. However, given the timescales over which transcription regulation takes place, the folding delays should not form the bottleneck in the experiments. We elaborate on potential improvements of the method in the reply to question 5.

4. Considering the paper focuses on transcription regulation in an in vitro setting, how do the authors envision the application of their findings in more complex in vivo systems? What are the potential challenges and opportunities in translating these quantitative insights to understand transcription regulation in a cellular context?
Reply: As is true for all in vitro studies, the environment of a cell consists of a vast number of components that are missing in vitro. What the influence of the difference in complexity is, must indeed be addressed when novel transcriptional scenarios are studied. Direct translation to more complex in vivo systems is beyond the focus of this paper, although our main findings are in agreement with previous in vivo work. In future work we plan to extend the procedure to cells.

5. How do the authors believe their findings could influence the design of future experimental studies in gene regulation? Are there particular aspects of their methodology that could be adapted or improved by other researchers to explore gene expression dynamics more comprehensively?
Reply: In future studies, (synthetic) genetic circuits could be analyzed in vitro, complementary to in vivo experiments. Having both in vitro and in vivo techniques to probe a system allows to study different variables in vitro to understand behavior in vivo. We think that improvements in the specific Broccoli sequence and associated dyes could enhance sensitivity and potentially reduce folding times. There has been some notable progress in recent years in tailoring of Broccoli-binding dyes for specific applications. For instance, a variation of BI, an improved version of DFHBI-1T, has been synthesized that has a faster fluorophore recycling rate, thereby offering increased resistance to photobleaching. This might enable quantitative in vivo experiments using Broccoli, as this was previously difficult to attain due to photobleaching. Regarding the Broccoli sequence, future studies could use 2x dimeric Broccoli instead of 2x Broccoli, which might lead to enhanced sensitivity, enabling the exploration of scenarios with even lower transcriptional activity.

Changes in manuscript: added to Discussion “In recent years, improvements have been made to Broccoli-binding fluorophores, enabling higher sensitivity and in vivo cell imaging.(Li et al. 2020, Li et al. 2021) Use of these dyes, possibly in combination with Broccoli variants that bind more fluorophores, might improve the method in future studies.”

Others:

Figure resolutions: The main text figures need to have their resolutions increased to match the clarity of the supplementary figures (SFigs).

Line 77 and 79: Replace the incorrect front quotation mark in "adsorbed" with the correct quotation mark.

Line 212: Correct the reference to Fig4B, which does not exist in the manuscript. This should be updated to refer to the correct figure based on the context provided in the manuscript.

Line 217: Change "Fig 4" to "Fig 3" to match the correct figure referenced in the text.

Line 244: Change "Fig 3" to "Fig 4" to correct the figure reference according to the context in the manuscript.

The "o" in the caption for Fig 5 seems to be a typo or an unclear abbreviation. The sentence should likely read, "The fraction of DNA bound to..." without the "o," unless "o" is intended to denote something specific.

Reply: We thank the reviewer for pointing out these errors and implemented all the suggestions.

**Reviewer #4**: The recent development of Broccoli and similar RNA aptamers has great potential to visualize RNA biology in real time, and is smaller or more direct than MS2-based systems and those requiring mRNA to be translated to reporter proteins. In this manuscript, the authors have employed Broccoli to quantify numerous aspects of transcription in vitro. These include free energies of specific and non-specific (NS) binding to DNA, the impacts of NS DNA or protein repressors, and kinetics of the reporter itself. Generally speaking, the formulas and logic to their derivation, as well as the experiments themselves, are well-performed. The use of a nearly minimal system is a plus and the 10-30s pulse between fluorescent measurements is indeed above the photobleaching recovery time published by others, though it may be worth commenting if varying the recovery time has any general effects on such readouts.

Reply: We thank the reviewer for their positive remarks. We have not observed any effect when varying the recovery time. We do not expect fluorophore bleaching to have a major effect in this in vitro setup, as the fluorescence is only measured in the center of the well, compared to experiments where large areas of cells are irradiated and the recovery time is indeed a factor to take into account.

Changes in manuscript: added to Discussion: “Photobleaching of the fluorescent Broccoli complex has also formed a constraint in studies that use Broccoli as a visualization tool to study transcription dynamics in live cells. In our system, we do not expect fluorophore bleaching to have a major effect, as the fluorescence is only measured in the center of the well. This leaves a significant part of the reaction volume unaffected by irradiation, allowing for efficient replacement of bleached molecules. However, in recent years, improvements have been made to Broccoli-binding fluorophores, enabling higher sensitivity and in vivo cell imaging.(Li et al. 2020, Li et al. 2021) Use of these dyes, possibly in combination with Broccoli variants that bind more fluorophores, might improve the method in future studies**.**”

The Broccoli aptamer folding into a relatively simple secondary RNA structure, or associated binding of DFHBI-IT, having such a long time to reach an excitable conformation is rather intriguing. While it would be helpful to measure the kinetics the compound binding to Broccoli that has had time to fold, work done on the Baby Spinach aptamer and DFHBI would indicate that this indeed is likely to be orders of magnitude more rapid and unlikely to effect the conclusions.

Reply: The binding of the fluorophore to the RNA is indeed orders of magnitude more rapid, as measured by Li et al. (2020).

Changes in manuscript: added in section Folding delays: “In more recent work by Purhonen *et al.* (2024)*,* a similar delay in the fluorescent signal can be observed in their transcription experiments.”

The relatively close free energies of binding to the promoter-driven DNA and non-specific DNA is also an interesting finding.

The PLOS Data Policy may require the plotted values and errors as supplementary tables or available in an online repository.
Reply: Yes, we will make the data available as supplementary material.

The temperature of the in vitro transcription should be specified (37°C?), for frame of reference for the kinetics.
Reply: Thanks for pointing out this omission; we will add the temperature (34°C) to the Methods section.

Changes in manuscript: added to sentence in Methods, In vitro transcription: “The fluorescent signal was then measured for 1 -2 hours **at 34°C** using a plate reader […]”

Minor text comments:
Figures 5 and 6 are better labelled as supplementary figures.

In line 137, it would be easier to read if the 2 and 3 in Cl2/(HCl)3 were made subscript

The section for "Repression using LacI" quotes Fig. 4 in the text when it should be Fig. 3.

Line 361: capitalize figure

Figure 5 legend: remove the o in "o of DNA"

Reply: We implemented all minor text comment suggestions.

6. PLOS authors have the option to publish the peer review history of their article ([what does this mean?](https://journals.plos.org/plosone/s/editorial-and-peer-review-process#loc-peer-review-history)). If published, this will include your full peer review and any attached files.


If you choose “no”, your identity will remain anonymous but your review may still be made public.


**Do you want your identity to be public for this peer review?** For information about this choice, including consent withdrawal, please see our [Privacy Policy](https://www.plos.org/privacy-policy).

Reviewer #1: No

Reviewer #2: No

Reviewer #3: No

Reviewer #4: **Yes:** Adam W. Whisnant
